# Biosynthesis of Silver and Gold Nanoparticles and Their Efficacy Towards Antibacterial, Antibiofilm, Cytotoxicity, and Antioxidant Activities

**DOI:** 10.1007/s12010-022-04199-7

**Published:** 2022-11-07

**Authors:** Mohamed K. Y. Soliman, Salem S. Salem, Mohammed Abu-Elghait, Mohamed Salah Azab

**Affiliations:** Botany and Microbiology Department, Faculty of Science, Al-Azhar University, 11884 Nasr City, Cairo, Egypt

**Keywords:** Green synthesis, Silver nanoparticles, Gold nanoparticles, Antibiofilm, Cytotoxicity, Antioxidant activity

## Abstract

The World Health Organization (WHO) reports that the emergence of multidrug-resistant and the slow advent of novel and more potent antitumor and antimicrobial chemotherapeutics continue to be of the highest concern for human health. Additionally, the stability, low solubility, and negative effects of existing drugs make them ineffective. Studies into alternative tactics to tackle such tenacious diseases was sparked by anticancer and antibacterial. Silver (Ag) and gold (Au) nanoparticles (NPs) were created from *Trichoderma saturnisporum*, the much more productive fungal strain. Functional fungal extracellular enzymes and proteins carried out the activities of synthesis and capping of the generated nano-metals. Characterization was done on the obtained Ag-NPs and Au-NPs through UV–vis, FTIR, XRD, TEM, and SEM. Additionally, versus methicillin-sensitive *Staphylococcus aureus* (MSSA) and methicillin-resistant *Staphylococcus aureus* (MRSA), *Pseudomonas aeruginosa*, and *Klebsiella pneumoniae*, the antibacterial activities of Ag-NPs and Au-NPs were assessed. In particular, the Ag-NPs were more effective against pathogenic bacteria than Au-NPs. Furthermore, antibiofilm study that shown Au-NPs had activity more than Ag-NPs. Interestingly, applying the DPPH procedure, these noble metallic NPs had antioxidant activity, in which the IC_50_ for Ag-NPs and Au-NPs was 73.5 μg/mL and 190.0 μg/mL, respectively. According to the cytotoxicity evaluation results, the alteration in the cells was shown as loss of their typical shape, partial or complete loss of monolayer, granulation, shrinking, or cell rounding with IC_50_ for normal Vero cell were 693.68 μg/mL and 661.24 μg/mL, for Ag-NPs and Au-NPs, respectively. While IC_50_ for cancer cell (Mcf7) was 370.56 μg/mL and 394.79 μg/mL for Ag-NPs and Au-NPs, respectively. Ag-NPs and Au-NPs produced via green synthesis have the potential to be employed in the medical industry as beneficial nanocompounds.

## Introduction

Antimicrobial resistance (AMR) is becoming a developing public health around the world and infections of healthcare-associated are difficult to be treated with many antibiotics [1]. AMR causes strong infections and lead to mortality and morbidity globally [2, 3]. Broad-based antibiotic therapy is required for a patient with multidrug-resistant germs; however, this therapy is time-consuming. In addition, antimicrobial resistance poses a serious threat across the world [4, 5]. According to WHO pandemic and epidemic diseases report, infection with drug-resistant pathogens features a higher death rate [6]. Resistance to traditional antibiotic behavior of bacteria related to Gram-positive or Gram-negative was due to many mechanisms such as efflux pumps of the cell to remove the antibiotics, changing of the site that antibiotics attached, and inactivation of antibiotics by enzymes secreted by the cell [7]. The bacteria that formed inside the biofilm matrix not only carry immunological responses but also have higher resistance to current antimicrobial treatments. Such a complex network increases the chance that bacteria may acquire resistance to a variety of medicines, making conventional antibiotic therapy ineffective [8]. High doses of antibiotics will be used to battle MDRO infections, which may cause unacceptable toxic and unpleasant effects, prompting the development of alternate therapies [9, 10]. The prevalence of breast cancer as well as the frequency of breast cancer-related death has decreased thanks to comprehensive detection and cutting-edge treatment techniques. Breast cancer is already thought of as a “chronic illness,” which acknowledges the significance of patients’ quality of life and also improved survival rates. Gene expression analysis with predictive or diagnostic value allows for individualized treatment through personalized therapy, bypassing chemotherapy in populations. Additionally, less invasive methods of controlling early-stage breast cancer now consider the patient’s appearances and reduce the long-term effects of lymphedema [11, 12].

We have seen huge changes because of nanotechnology’s revolutionary approach, with the birth of nanoparticles with distinct functions and size-dependent physiochemical features [13–18]. Nanomaterial’s research focuses on the creation of nanoparticles of diverse sizes, shapes, and structures for specific purposes [19–24]. Nanoparticle shape and size parameters are frequently varied by varying chemical concentrations and reaction conditions [25–29]. When the synthesized nanoparticles are used, they will encounter obstacles such as bioaccumulation, toxicity, modeling variables, regeneration, reuse, and recycling [30–32]. NPs have several applications, including drug delivery [33], antimicrobial [34–38], anti-cancer [39], antioxidant and catalytic activities [40–42], anticancer and anti-inflammatory activity [43], and hepatoprotective [44].

Ag-NPs and Au-NPs have sparked a lot of attention among noble metal nanomaterials as an entirely new antibacterial agent. They also exhibit outstanding properties like strong plasmon resonance; electrical, magnetic, and thermal conductivity; antibacterial, antiviral, and antimalarial action; and bio-stability [45]. Cancer diagnostics, photo-thermal therapy, bio-labeling, nano-diagnostics, drug delivery, gene transfer, immune chromatography, and pathogen detection in clinical samples have recently made considerable usage of Ag-NPs as well as Au-NPs [46, 47]. NPs were created in several methods, including physically, chemically, and biologically [48–53]. To produce NPs, chemical and physical techniques are the most prevalent. However, because these synthesis procedures include hazardous chemicals that are environmentally harmful, they are highly expensive and result in the development of poisonous by-products [54]. Even though physicochemical techniques are still quite costly, and it may generate toxic by-products, the biological method is recommended because of benefits like limited energy needs, non-toxic reagents, biocompatibility, ease of processing, enhanced stability, elimination of unnecessary processing during synthesis, and sustainability [55–57]. Organic biomolecules have a dual role in the biological synthesis of metal NPs, decreasing and stabilizing the NPs [58–61]. Biological agents such as bacteria, fungus, actinomycetes, yeast, and plants are used in the biological technique for NP synthesis [62, 63]. Biological agents produce many enzymes (metabolites) that cause metallic ions to be reduced enzymatically [64, 65]. The fungal-mediated green production of NPs provides a lot of advantages, including easy scaling up, processing, economic viability, biomass processing, and the recovery of significant surface distances with optimal mycelia outgrowth [66–68]. Furthermore, compared to other biological approaches, the use of fungal biomass filtrate containing various metabolites as a green strategy for the synthesis of metallic NPs is preferable.

This work produced and characterized Ag-NPs and Au-NPs by using the metabolites of *Trichoderma saturnisporum*. In order to exploit mycosynthesized Ag-NPs and Au-NPs as smart nanomaterials in the medical field, their antibacterial, antibiofilm, antioxidant, antitumor, and cytotoxic properties were investigated.

## Materials and Methods

### Materials

Silver nitrate (AgNO_3_) and chloroauric acid (HAuCl_4_) were purchased from Sigma-Aldrich, USA, for chemicals and were used as precursors for the preparation of Ag-NPs and Au-NPs. Other chemicals, culture media, and reagents used in this study were purchased from Modern Lab Co., India, in analytical grade without any purification required.

### Isolation and Identification of Fungal Isolate

The fungal strain *Trichoderma saturnisporum* was isolated from agriculture soil in Qalyub Governorate, Egypt. For isolation of fungal strain, potato dextrose agar (PDA) was used and incubated at 28 ± 2 °C for 3–4 days. In the molecular identification using genomic DNA as well as its region amplification, the first most efficient fungal strain was genetically identified. For the ITS-based sequencing, 0.1 g of fungal mycelium genomic DNA was extracted. The Gene Jet Plant Genomic DNA purification Kit (Thermo) #k0791 procedure has been used to extract the DNA. ITS1 and ITS4 have been the primers employed. The Maxima Hot Start PCR Master Mix (Thermo) #k0221 by Sigma Scientific Services Company (Cairo, Egypt) was used in the following amplification (PCR) procedure: Thermo’s Maxima Hot Start PCR Master Mix, 0.5 µM from each primer, and 1 µL of isolated fungal genomic DNA were all added to a 50µL PCR mixture. In a DNA Engine Thermal Cycler, the PCR was carried out with a hot start at 94 °C for 3 min, followed by 30 cycles of 94 °C for 30 s, 55 °C for 30 s, and 72 °C for approximately 60 s, then a further 10 min at 74 °C of extension. The samples were sequenced by GATC Company using an ABI 3730 1 DNA sequencer using forward and reverse primers (Germany). The ITS sequences of the detected fungal isolates were matched against the GenBank database.

### Biomass and Cell-Free Filtrate Preparation

Two disks of newly cultivated *Trichoderma saturnisporum* were added to 100 mL of Czapek-Dox broth (CDB) medium after the pH was adjusted to 6.8–7.2. The flasks were then placed inside a rotary orbital shaker and rotated at a speed of 120 rpm for 4 days at 32 °C. Following an incubation period, the biomass was removed by being passed through four layers of wool fabric. *Trichoderma saturnisporum*’s biomass was collected using filter paper No. 1, rinsed in sterilized distilled water to eliminate any remaining medium components, and then suspended in 100 ml distilled water. At 32 °C, the mixture was stirred for 72 h. The cell-free filtrate of *Trichoderma saturnisporum* was obtained and prepared for use in the creation of nanoparticles.

### Biosynthesize of Ag-NPs and Au-NPs by *Trichoderma saturnisporum*

The synthesis of Ag-NPs and Au-NPs utilized the cell-free filtrate in the manner described below. Before incubating with cell-free filtrate at 28 ± 2 °C for 72 h on an orbital shaker (150 rpm) in the dark, 1 millimole of AgNO_3_ and HAuCl_4_ was incubated separately and mixed with cell-free filtrate of *Trichoderma saturnisporum*, and the pH was then adjusted at 10 and 5 for Ag-NPs and Au-NPs, respectively. Following the incubation time, Ag-NPs and Au-NPs showed a brown and violet colors, respectively. The latter was separated and dried for 48 h at 150 °C. Finally, the Ag-NPs and Au-NPs products were collected and subjected for further investigation.

### Characterization of Silver and Gold NPs

Biosynthesis of Ag-NPs and Au-NPs colloids were routinely monitored using UV–vis spectra on JASCO V-630 UV-VIS Spectrophotometer (JASCO INTERNATIONAL CO., LTD) at wavelengths of 200–800 nm to detect intense absorption peak which related to surface plasmon excitation. Ag-NPs and Au-NPs that were synthesized from biological materials were measured using a transmission electronic microscope (TEM) (JEOL-2100). To do this, a drop of NP-containing solution was applied to coated-carbon copper grids that were then vacuum-dried for a whole night before being loaded into a specimen holder. Additionally, various functional groups present in bio-fabricated Ag-NPs and Au-NPs molecules were investigated by Fourier transform infrared (FTIR) spectroscopy (JASCO, FT/IR-6100). The NP samples were mixed with KBr and then compressed under intense pressure into disks. To get FTIR spectra, these disks were scanned between 400 and 4000 cm^−1^. Mycosynthesized Ag-NPs and Au-NPs’ surface morphology and elemental structures were investigated using SEM connected to a JEOL JSM-6510 LV energy dispersive spectroscopy (EDS) device. The crystal structures of Ag-NPs and Au-NPs were described by XRD analysis (XRD, X PERT PRO-PAN Analytical).

### Antimicrobial Activity

By using a broth microdilution technique, the MIC values of two various substances (Ag-NPs and Au-NPs) were tested against multidrug-resistant bacteria (MRSA, MSSA, *P. aeruginosa*, and *K. pneumonia*). Ag-NPs and Au-NPs were prepared at various concentrations. Achieve final concentrations of 1000, 500, 250, 125, 62.5, 31.25, and 15.75 µg/mL. Test samples (100 µL) of varying concentrations were put into sterile microtiter plate wells that were already filled with 100 µL of double-strength Mueller-Hinton (MH) broth. In all wells except the negative control well, bacterial cell suspension (20 µL) corresponding to OD equal to 0.5 McFarland standard was added. To test whether MH broth could adequately support bacterial growth, bacterial solution was added to positive control wells. The MH broth and sterile distilled water used in the negative control wells were used to ensure sterility. The plates were incubated for 24 h at 37 °C. After that, the plate subsequently re-incubated again for 6 h with 30 µL of resazurin solution (0.02% w/v) added to each well to detect bacterial growth. Bacterial growth was indicated by a shift in color from blue to red. Red growth control wells indicated that the strains were being grown appropriately, and no change in the color of a sterile control well suggested that there had been no contamination. The experiment is performed three times, and mean values were reported.

### In Vitro Biofilm Inhibition

The MTP method was used to evaluate the ability of Ag-NPs and Au-NPs to inhibit or reduce the biofilm aggregation of clinical species *S. aureus* and *pseudomonas* spp*.* (identified as a potent biofilm-producing strain) with some modifications [69]. Briefly, gradient concentrations of the Ag-NPs and Au-NPs were distributed into a flat-bottomed MTP containing tryptic soy broth media (TSB) with 1% glucose. Overnight culture was performed of test organisms diluted into 1:100 to reach an inoculum size 1.5 × 10^8^ CFU/mL then loaded onto MTP and incubated at 37 °C for 48 h. Growth density was measured spectrophotometrically (OD 620 nm) after the planktonic cells removed from all of wells of MTP without any disruption of the formed biofilm. Furthermore, to remove the residue cells of floated unbounded cells, these wells were washed with phosphate-buffered saline (PBS) at pH 7.4, three times. For fixation of biofilm, 95% methanol was added in equal volume of 200 μL to all wells. After that, add 200 μL of 0.3% crystal violet (CV) to the same wells and then incubate the plates at 20–25 °C for 15 min. Additionally, the excess of CV stain was gently removed by sterile distilled water. Lastly, CV stain bounded with biofilm at this point was examined and then photographed using an inverted microscope (Olympus Ck40) × 150. For quantitative biofilm formation detection, add 200 μL of 30% acetic acid to all wells, and the microplate reader (Tecan Elx800) was used for measuring the color at 540 nm. The results of the treated wells and untreated were compared.

### Antioxidant Activity

Antioxidant activity of Ag-NPs and Au-NPs was carried out using the DPPH (2,2-diphenyl-1-picrylhydrazyl) method as previous study with minor modifications [55]. Different concentrations of nanoparticles (1000, 500, 250, 125, 62.5, 31.25, and 15.62 µg/mL) were used to determine the scavenging of DPPH radicals. Antioxidant activity of standard and NPs was determined as DPPH scavenging activity. The equation below was used to determine antioxidant activity:

$$\mathrm{Antioxidant}\;\mathrm{activity}\left(\%\right)=\frac{\mathrm{Control}\;\mathrm{absorbance}-\mathrm{Sample}\;\mathrm{absorbance}}{\mathrm{Control}\;\mathrm{absorbance}}\times100$$

### Cytotoxic and Anticancer Activity

#### Cell Culture

The human mammary gland, breast, derived from metastatic site; adenocarcinoma cells (Mcf7-HTB-222); and normal Vero cells (kidney of African green monkey) were procured from ATCC.

#### MTT Assay

A full monolayer sheet formed after 24 h of incubation at 37 °C with 1 × 10^5^ cells/mL (100 μL) in the 96-well tissue culture plate. After a confluent sheet of cells had grown, the 96-well microtiter plates’ growth material was decanted, and the cell monolayer had been rinsed twice with washing media. In RPMI medium containing % serum, two-fold serial dilution of the sample was created (maintenance medium). Three wells served as the control wells and received just maintenance medium while 0.1 ml of each dilution was tested in various wells. The plate was tested after 37 °C of incubation. The physical characteristics of cytotoxicity, such as problems such as loss of the monolayer, rounded, shrinking, or cellular granulation, were examined in the cells. MTT solution (5 mg/mL in PBS) has been prepared (BIO BASIC CANADA INC). To every well, 20 μL of the MTT solution was then added. To completely blend the MTT into medium, put it on a shaker and shake for 5 min at 150 rpm. Permit the MTT to metabolize for 4 h in an incubator (37 °C, 5% CO_2_). In 200 μL of DMSO, re-suspend formazan (MTT metabolic product). For 5 min, shake at 150 rpm to properly combine the formazan and solvent. At 620 nm, remove background when reading optical density. Cell number and optical density ought to be closely connected.

#### Analytical Statistics

For all of the results obtained, the means of three replications and standard deviation (SD) were determined, and the data were subjected to analysis of variance means using the SigmaPlot 12.5 software.

## Results and Discussion

### Isolation and Identification of Fungal Isolate

**Fig. 1:**
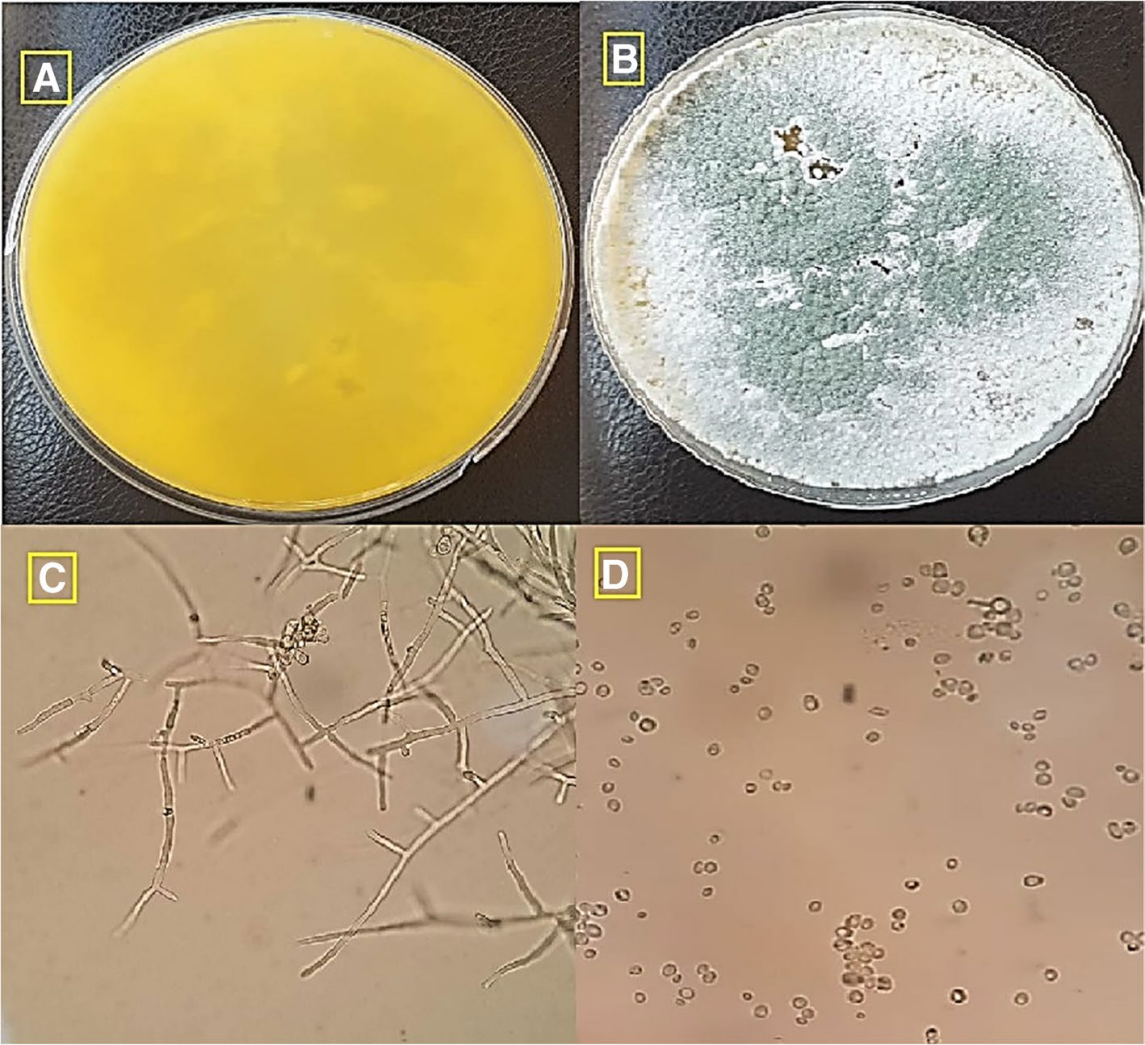
Morphological identification of *Trichoderma *sp. **A** Surface of culture on PDA. **B** Reverse color of culture. **C** Conidiophore and head under the light microscope (400×). **D** Conidia under the light microscope (800×)

The most potent microorganism in the soil is fungi, which have a good potential for bio-fabricated of metallic nanoparticles. In the current study, the fungal isolate has synthesized strong stable Ag-NPs and Au-NPs within 6 h. Then, this fungal isolate was subjected to further identification based on cultural characteristics and microscope examination and found that the fungal isolate followed the *Trichoderma* species according the manual identification and named *Trichoderma *sp. (Fig. 1). *Trichoderma* spp. were identified as suitable isolate for the extracellular manufacture of metallic nanoparticles in this study based on particle stability and quicker rate of synthesis. In a similar fashion, many researchers have also utilized *Trichoderma* spp. as promising candidates for either Ag-NPs or Au-NPs synthesis [70–73].

### Molecular Identification of *Trichoderma *sp.

**Fig. 2:**
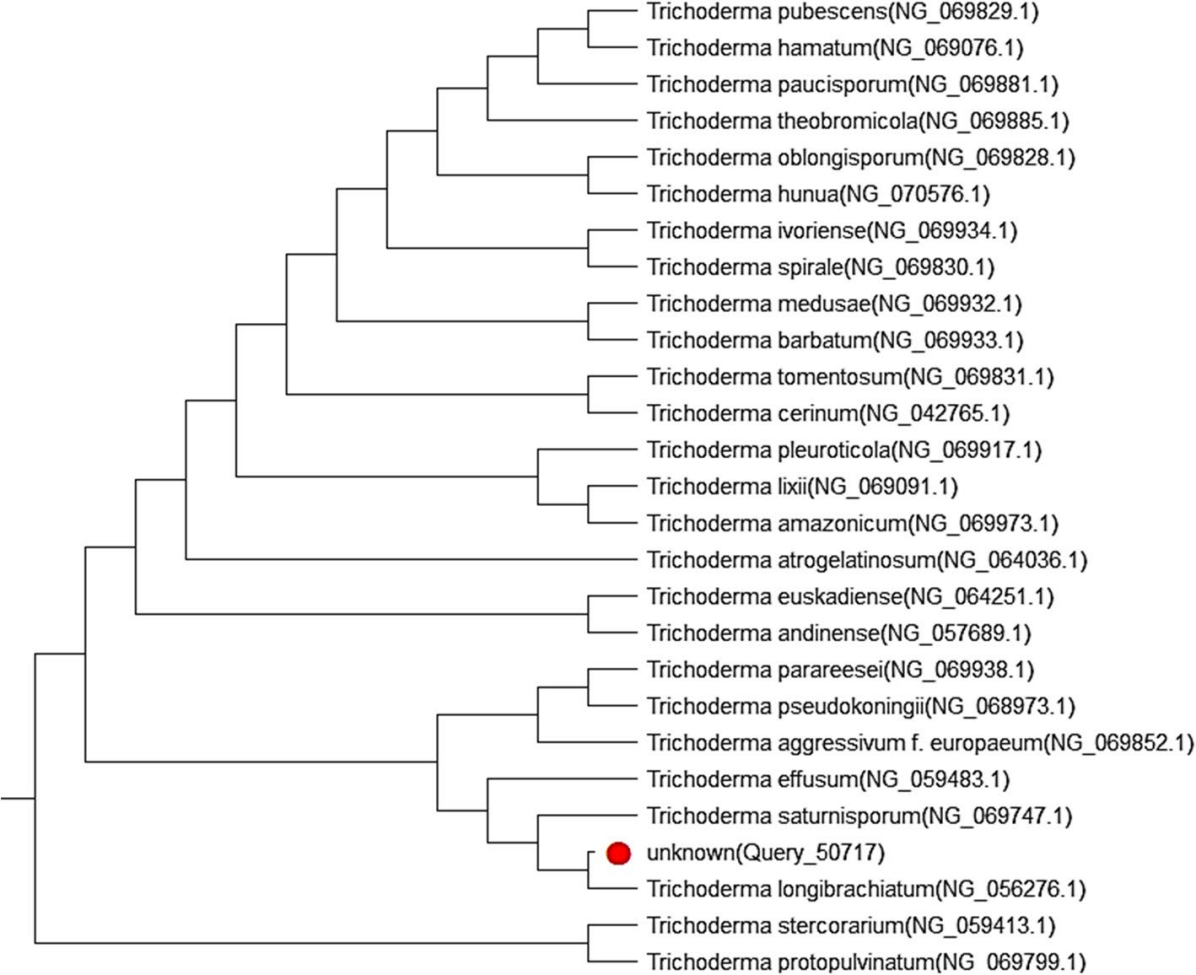
Phylogenetic tree incorporating the fungal isolate ITS sequences matching NCBI sequences, identified as *Trichoderma saturnisporum*

The fungus isolate *Trichoderma *sp. was identified by the molecular method. Fungal ITS fragment was identified by PCR and sequencing approaches (amplification and sequencing of ITS region have resulted in approximately 600 bp). We constructed a maximum-likelihood phylogenetic tree to correlate our ITS sequence with the previously described sequences (Fig. 2); the results showed that the sequenced ITS fragment was related to the topology of *Trichoderma saturnisporum* with a similarity of 99.43%.

### Biosynthesis and Characterization of Ag-NPs and Au-NPs

**Fig. 3:**
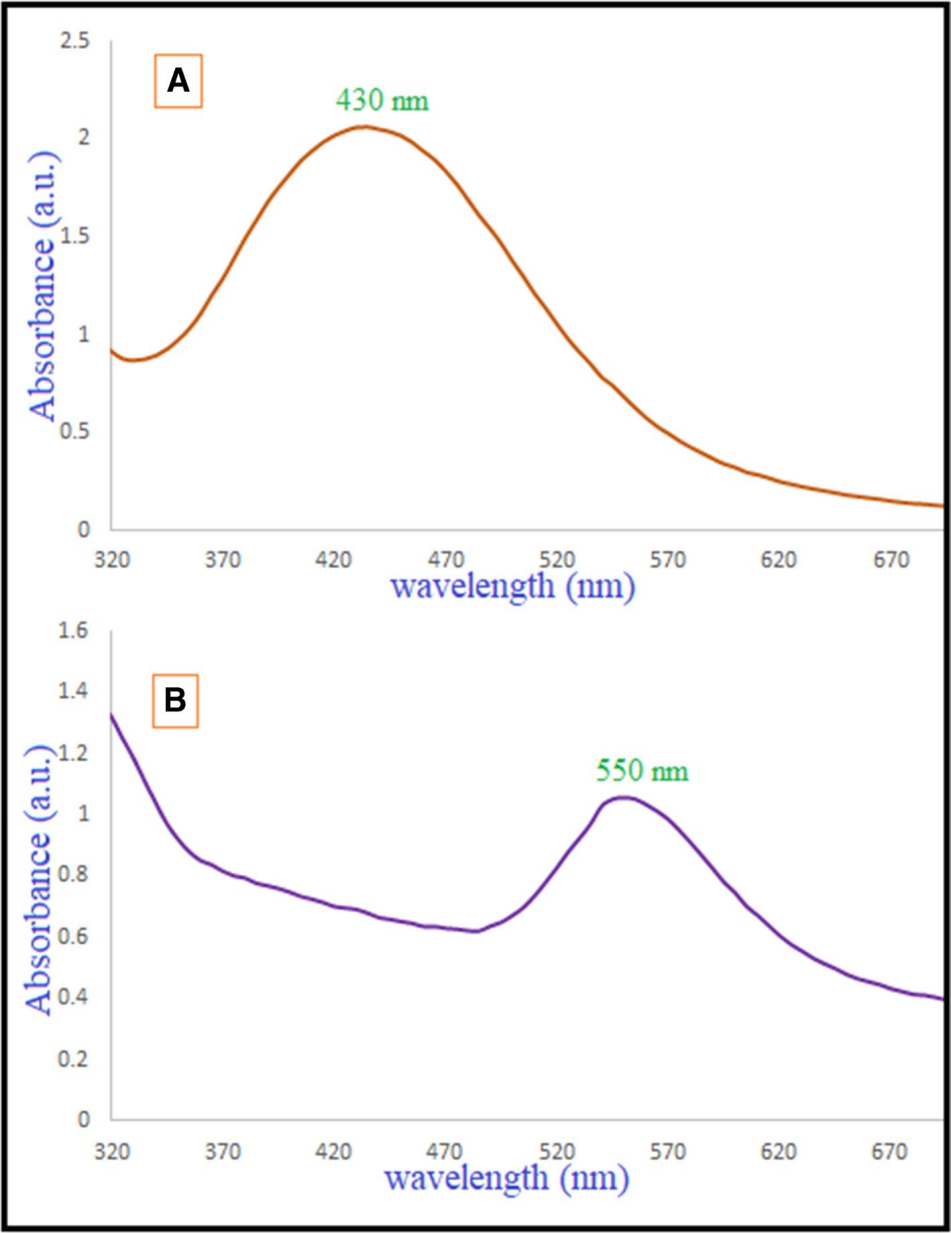
Absorption peaks of the mycosynthesized Ag-NPs (**A**) and Au-NPs (**B**) using a UV–vis spectrophotometer

*Trichoderma* is a well-established genus that can secrete a variety of extracellular enzymes and metabolites in enormous quantities, making it an attractive choice for the manufacture of metal nanoparticles on an industrial scale [74, 75]. The formation of Ag-NPs and Au-NPs was first detected by a change in color, turning brown for Ag-NPs and pinkish violet for Au-NPs, respectively. The UV–vis spectrum provided more evidence of its fabrication. UV–vis spectroscopy refers to absorption spectroscopy in the UV–vis region ranging from 300 to 800 nm. Noble metallic NPs like gold and silver possess wavelength of maximum absorption (*λ* max) ranging from 500 to 550 nm and 400 to 450 nm, respectively. Therefore, when we observe the *λ* max value in these wavelength regions, we can confirm that the corresponding NPs have been formed. As time progresses, there is a possibility of aggregation of NP which results in a shift of *λ* max towards the longer wavelength region. In Fig. 3, UV–vis spectra absorption peak at 430 and 550 nm verified the creation of Ag-NPs and Au-NPs, respectively.

**Fig. 4:**
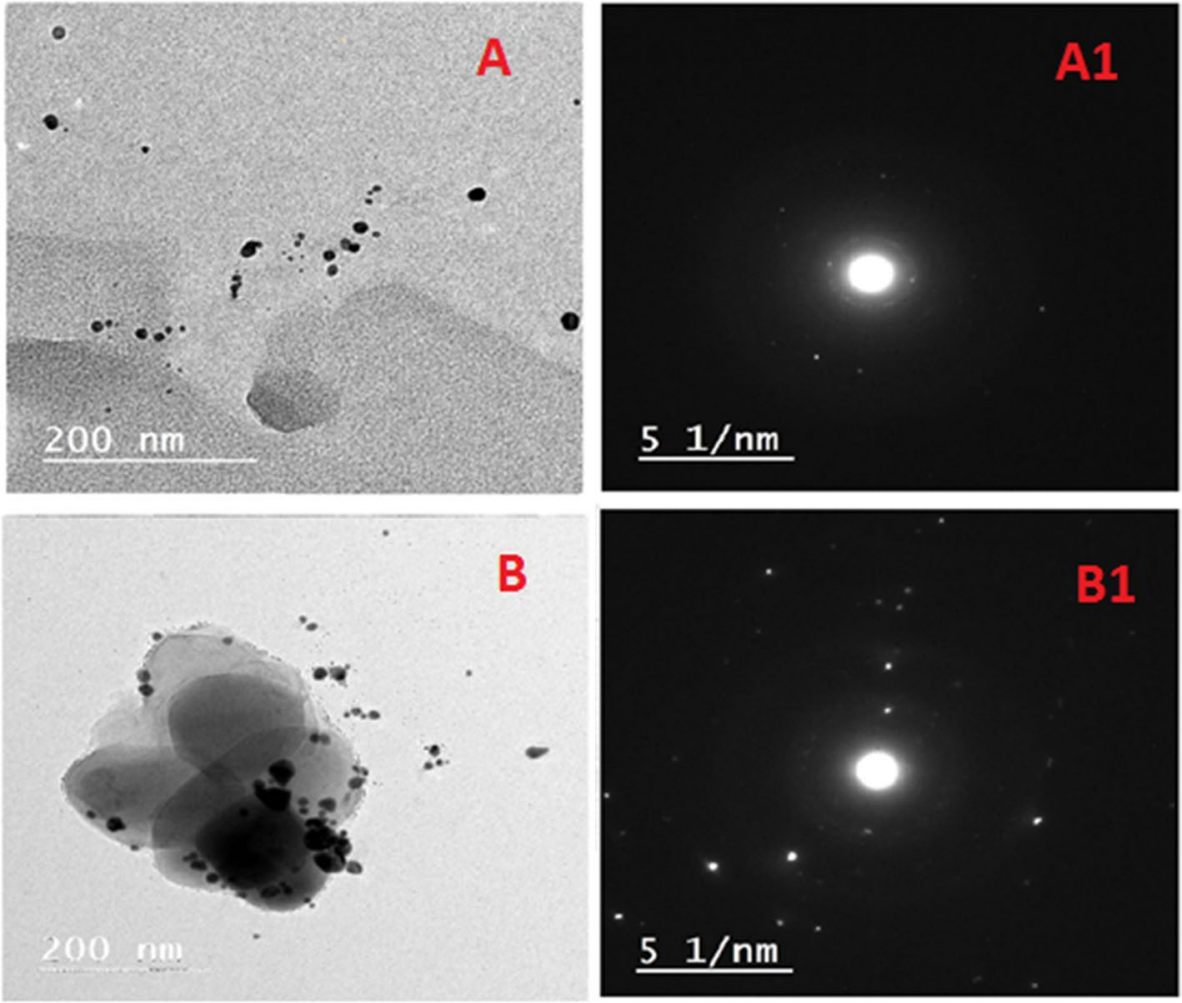
TEM images of Ag-NPs (**A**, A1) and Au-NPs (**B**, B1)

The TEM picture demonstrated nearly spherical shaped, well-dispersed Ag-NPs (Fig. 4A), as well as the focus displayed the SAED pattern (Fig. 4A1). The size of the particles was revealed in the range of 10–70 nm for Ag-NPs (Fig. 4A). El-Wakil synthesized spherical Ag-NPs from *Tricoderma *sp. that ranged in size from 40 to 80 nm [76]. Additionally, Ag-NPs produced by *Bacillus* sp. were discovered to be spherical, hexagonal, and triangular [77]. In contrast, Au-NPs were spherical and 8–30 nm in size (Fig. 4B). Interestingly, the SAED pattern of Au-NPs was also seen in Fig. 4B1. *Trichoderma* sp. was shown to synthesize spherical, polygonal, and triangular Au-NPs [73]. According to the procedures and microbe extracts used, the NPs’ size and form changed [78–80].

**Fig. 5:**
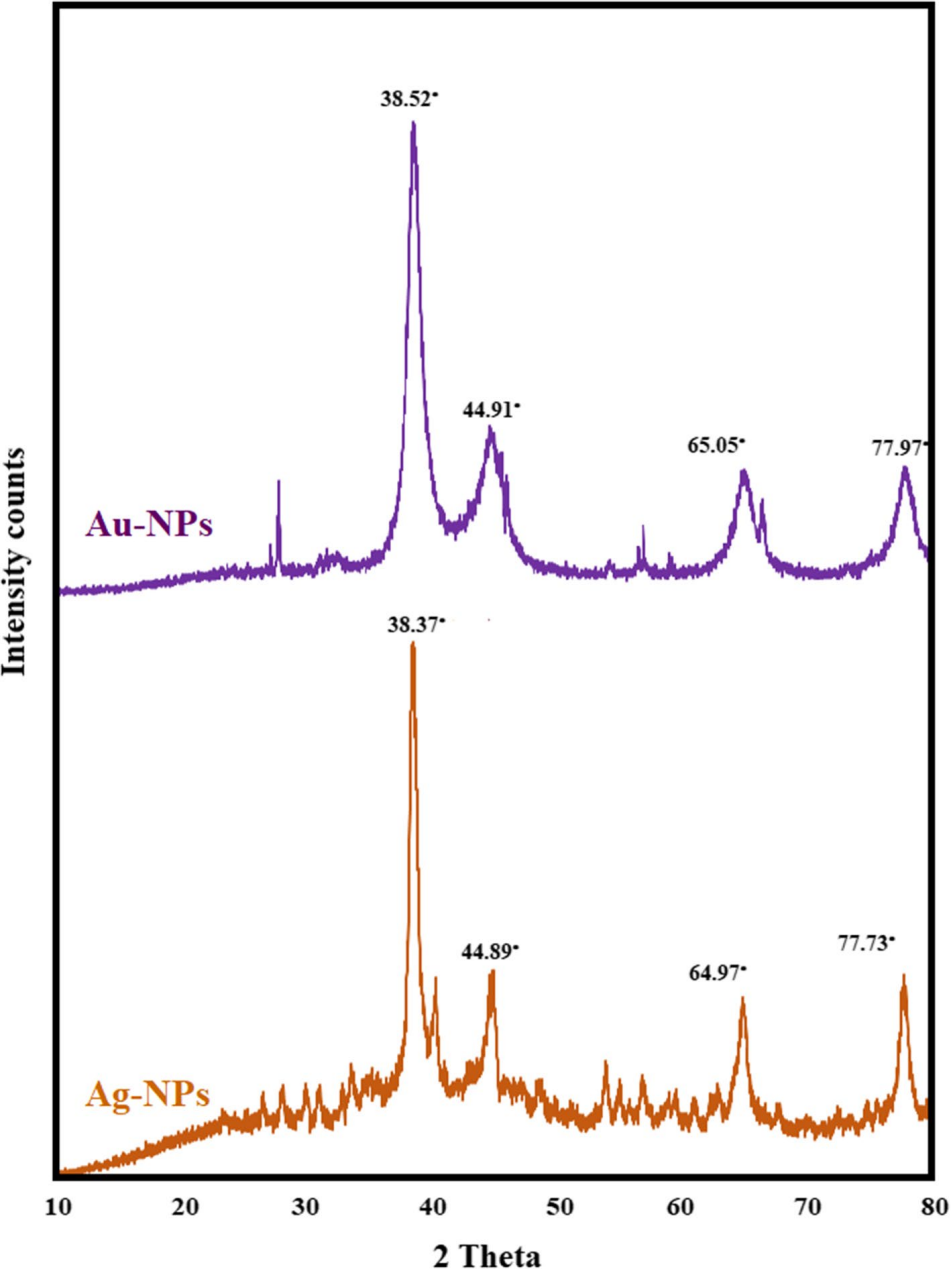
XRD pattern of Ag-NPs and Au-NPs biosynthesized by *Trichoderma saturnisporum*

Figure 5 shows the XRD patterns of Ag-NPs and Au-NPs. Four different diffraction peaks may be seen in each spectrum. Peaks are seen in the case of Ag-NPs at four values: 38.37°, 44.89°, 64.97°, and 77.73°. Peaks throughout Au-NPs were shown at 2*θ* values: 38.52°, 44.91°, 65.05°, and 77.97°. These 4 peaks correspond to reflections from of the planes (111), (200), (220), and (311) of a face-centered cubic phase of Ag-NPs and Au-NPs. The peak matching to the (111) plane is much stronger in both situations than the peaks belonging to the (200), (220), and (311) planes. This implies that perhaps the nanoparticles are mostly oriented throughout the (111) planes. Consequently, the XRD investigation obviously demonstrates that the produced Ag-NPs and Au-NPs are crystalline. These findings are consistent with previous research [78, 81].

**Fig. 6:**
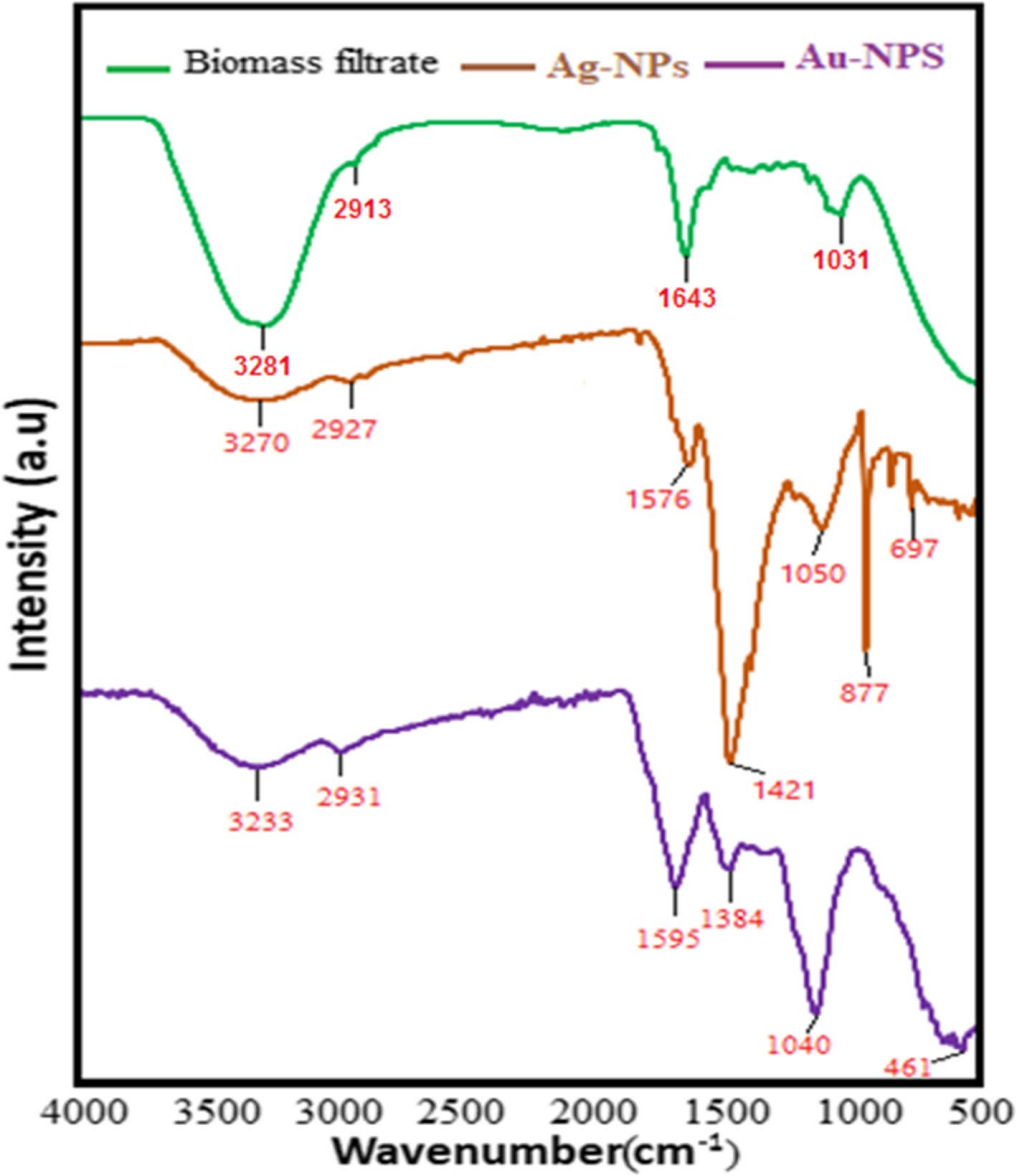
FTIR spectra of fungal filtrate, Ag-NPs, and Au-NPs biosynthesized by *Trichoderma saturnisporum*

FTIR analysis was done on the extract of the strain *Trichoderma saturnisporum* and the samples of biogenic NPs in order to look into the functional groups on the surface of the bio-NPs. The stretching vibration of the O–H or N–H bond was identified as the source of the strong stretching at 3281–3233 cm^−1^ in the FTIR spectrum (Fig. 6) of the extract and biogenic NPs. Possible correlations between the two bands at 2913–2931 cm^−1^ are C-H stretching vibration. It is appropriate to identify the amide I and amide II of polypeptides or proteins as the bands detected around 1643 and 1576 cm^−1^. The N–H stretched resonant seen in the amine groups of the proteins was responsible for the spectra at 1384 and 1421 cm^−1^. Aliphatic amines’ C-N stretching was identified as the cause of the bands at around 1040–1050 cm^−1^. The peaks in the region between 400 and 700 cm^−1^ are attributed to metal–oxygen vibration. In this study, the formation of silver nanoparticles can be confirmed by the presence of a peak at 697 and 877 cm^−1^ belonging to the bending vibration of Ag–O. Additionally, Au-O was assigned the characteristic band at 461 cm^−1^. The occurrence of those same peaks with a little variance in the wavenumber has previously been described [72, 82, 83].

**Fig. 7:**
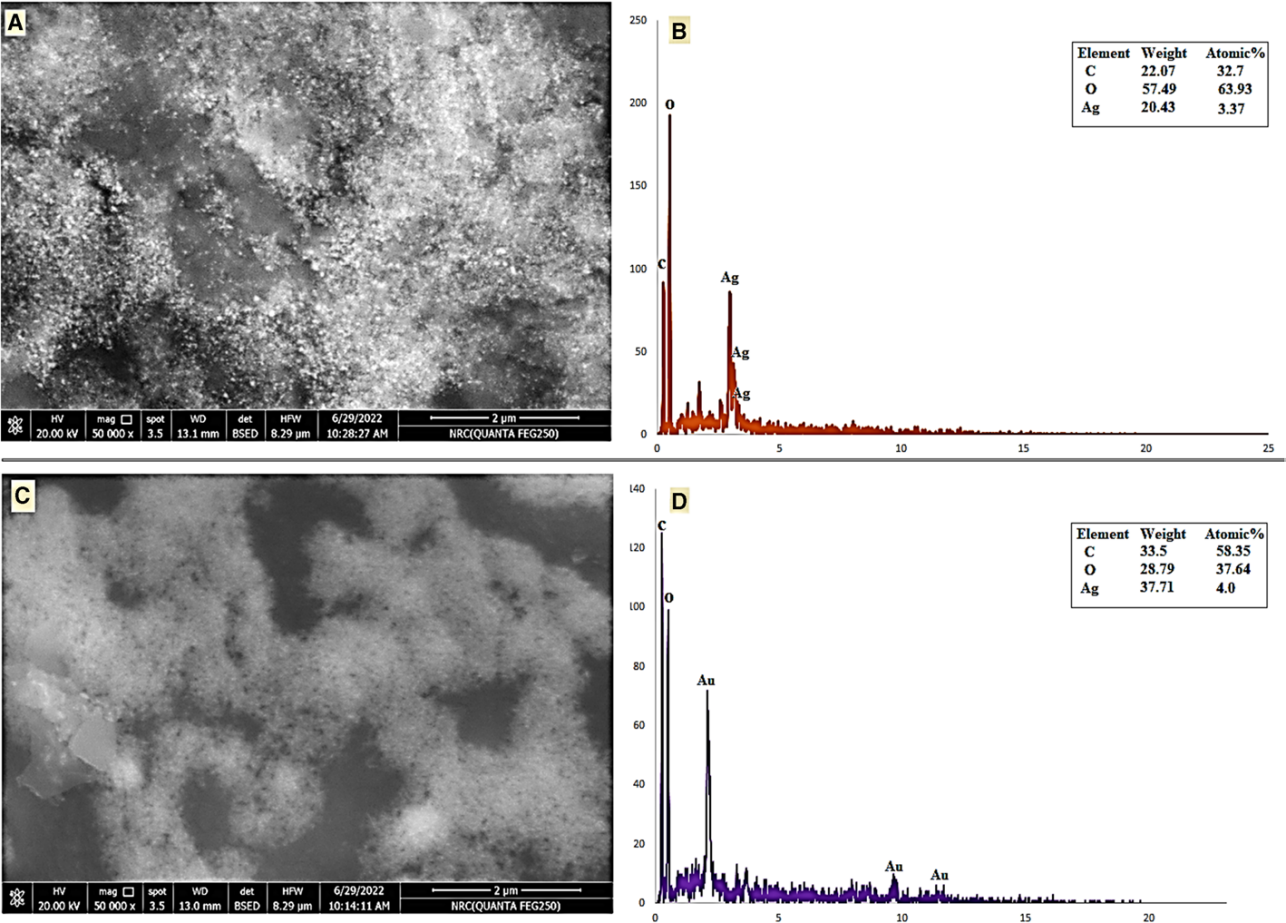
SEM–EDX analysis of mycosynthesized Ag-NPs and Au-NPs. **A** SEM image of Ag-NPs. **B** EDX spectrum of Ag-NPs. **C** SEM image of Au-NPs. **D** EDX spectrum of Au-NPs

SEM–EDX analysis was used to evaluate the surface morphology and quantity of both silver and gold (Fig. 7A–D). The Ag-NPs and Au-NPs were discovered to really be crystalline exhibiting spherical shapes (Fig. 7 A and C). SEM examination normally provides an in-depth image resolution of something like the particles by assigning a focused beam of electrons over the surface and finding secondary or back-scattered electron signal. The existence of the metals Ag and Au was substantiated by EDX spectrum analysis, which exhibited peaks within their typical energy levels. The identification lines presented for the principal emission energies for Ag correlate to the peaks observed in the spectrum, providing confidence that Ag was accurately characterized wherein the peak situated approximately 3 and 4 keV. Those maxima are directly related to the Ag characteristic (Fig. 7B). Similarly, an absorption peak was obtained at ∼2 keV which is specifically related to the characteristic of Au-NPs obtained in the spectrum (Fig. 7D). The mass of Ag was 20.43% with an atom of 3.37% whereas for gold, it was 37.71% with an atom of 3.0%. The EDX findings were remarkably consistent with other publications on the characterization of Ag-NPs and Au-NPs [39, 78, 84].

### Minimum Inhibitory Concentration of Ag-NPs and Au-NPs

**Fig. 8:**
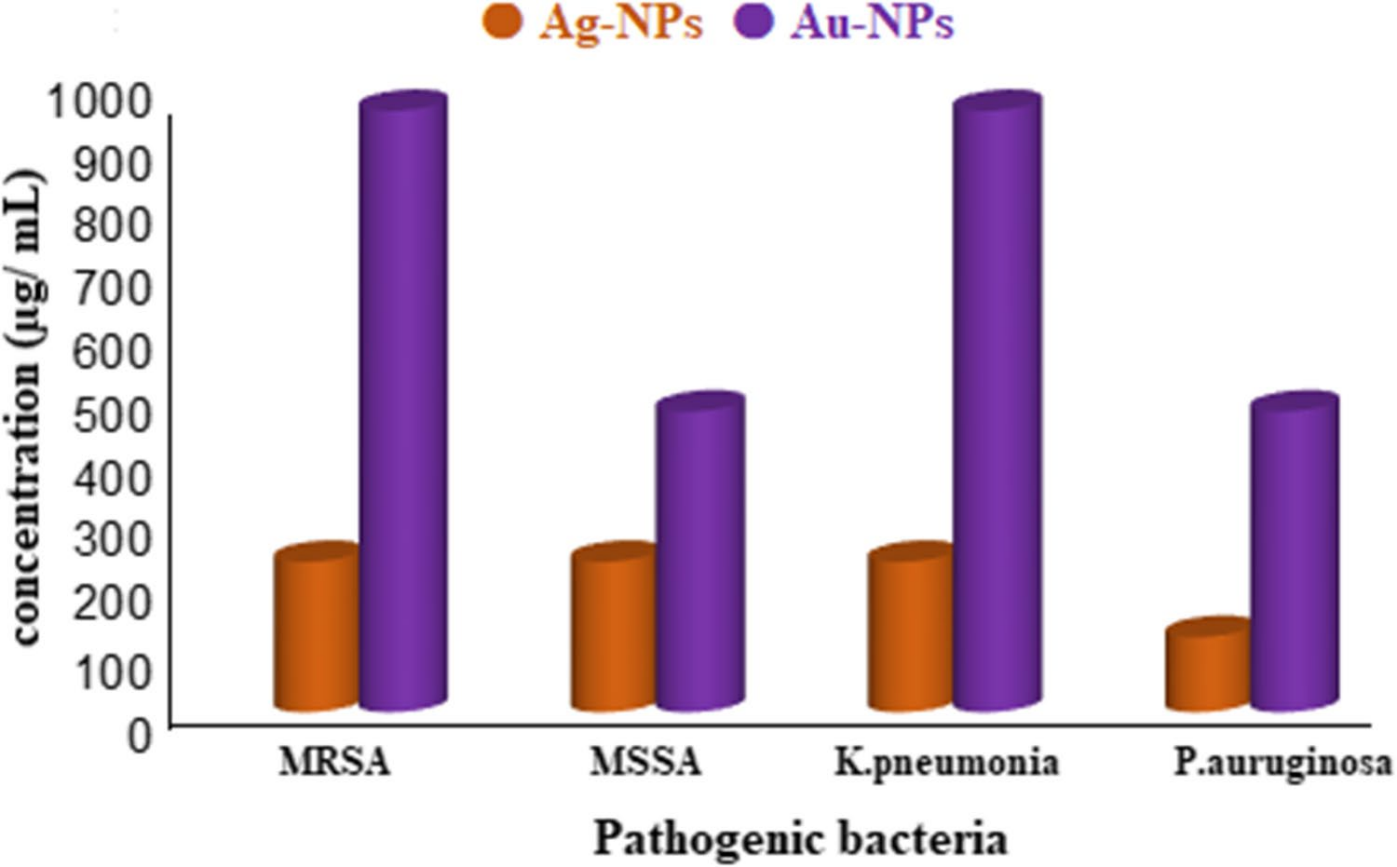
Antibacterial activity of Ag-NPs and Au-NPs against different pathogenic bacteria

One of the great importance of Ag-NPs and Au-NPs is their activities against pathogenic microbes. Therefore, mycosynthesized Ag-NPs and Au-NPs were firstly investigated for their antibacterial activity versus both pathogenic Gram-positive (MRSA and MSSA) and Gram-negative (*P. aeruginosa* and *K. pneumonia*) bacteria. The inhibitory effect of different concentrations of Ag-NPs and Au-NPs (16.62–1000 µg/mL) was investigated. Results showed that the MIC for Ag-NPs was 250 µg/mL against MRSA, MSSA, and *K. pneumoniae* and 125 µg/mL against *P. aeruginosa*, while the MIC for Au-NPs was 1000, 500, 1000, and 500 µg/mL against MRSA, MSSA, *K. pneumoniae*, and *P. aeruginosa* respectively (Fig. 8). Furthermore, Ag-NPs were more effective than Au-NPs against Gram-positive and Gram-negative pathogenic bacteria. Another research revealed that nanoparticles reduce bacterial growth by interacting with phosphorous moieties in DNA, which leads to inactivation of DNA replication and therefore decrease of enzyme activity [36, 38, 85]. Also, it can inhibit respiratory enzymes of bacterial cells and stop ATP production which leads to cell death. Besides, electrostatic attractions between positive charge of NPs and negative charge of bacterial surface led to several changes as membrane detachment, cytoplasmic shrinkage, and ultimately membrane of cell rupture [86, 87].

### Anti-biofilm of Ag-NPs and Au-NPs

**Fig. 9:**
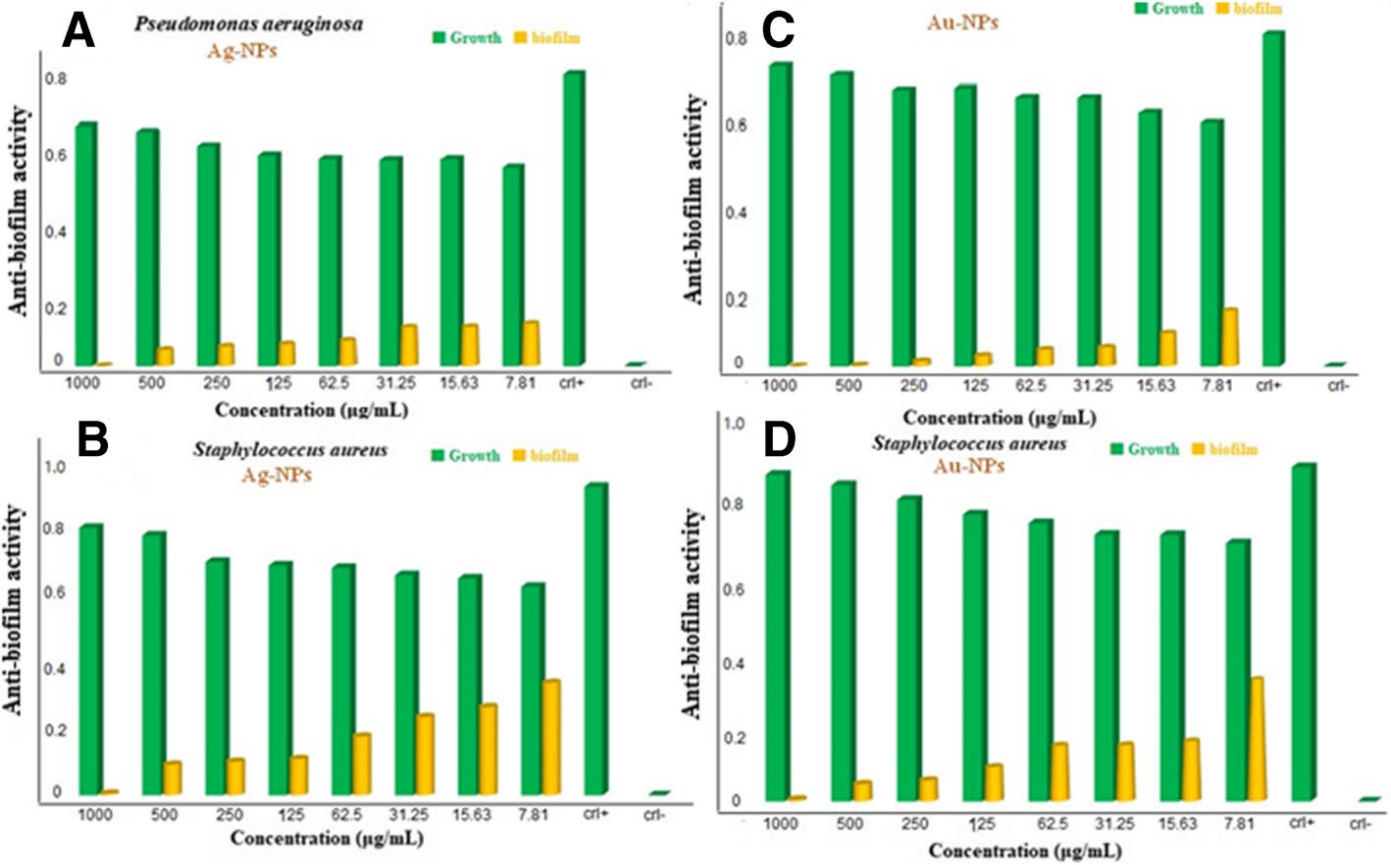
Anti-biofilm activity of the Ag-NPs and Au-NPs against Gram-positive and Gram-negative bacteria. **A** Ag-NPs against *Pseudomonas aeruginosa*. **B** Ag-NPs against *Staphylococcus aureus*. **C** Au-NPs against *Pseudomonas aeruginosa*. **D** Au-NPs against *Staphylococcus aureus*

**Fig. 10:**
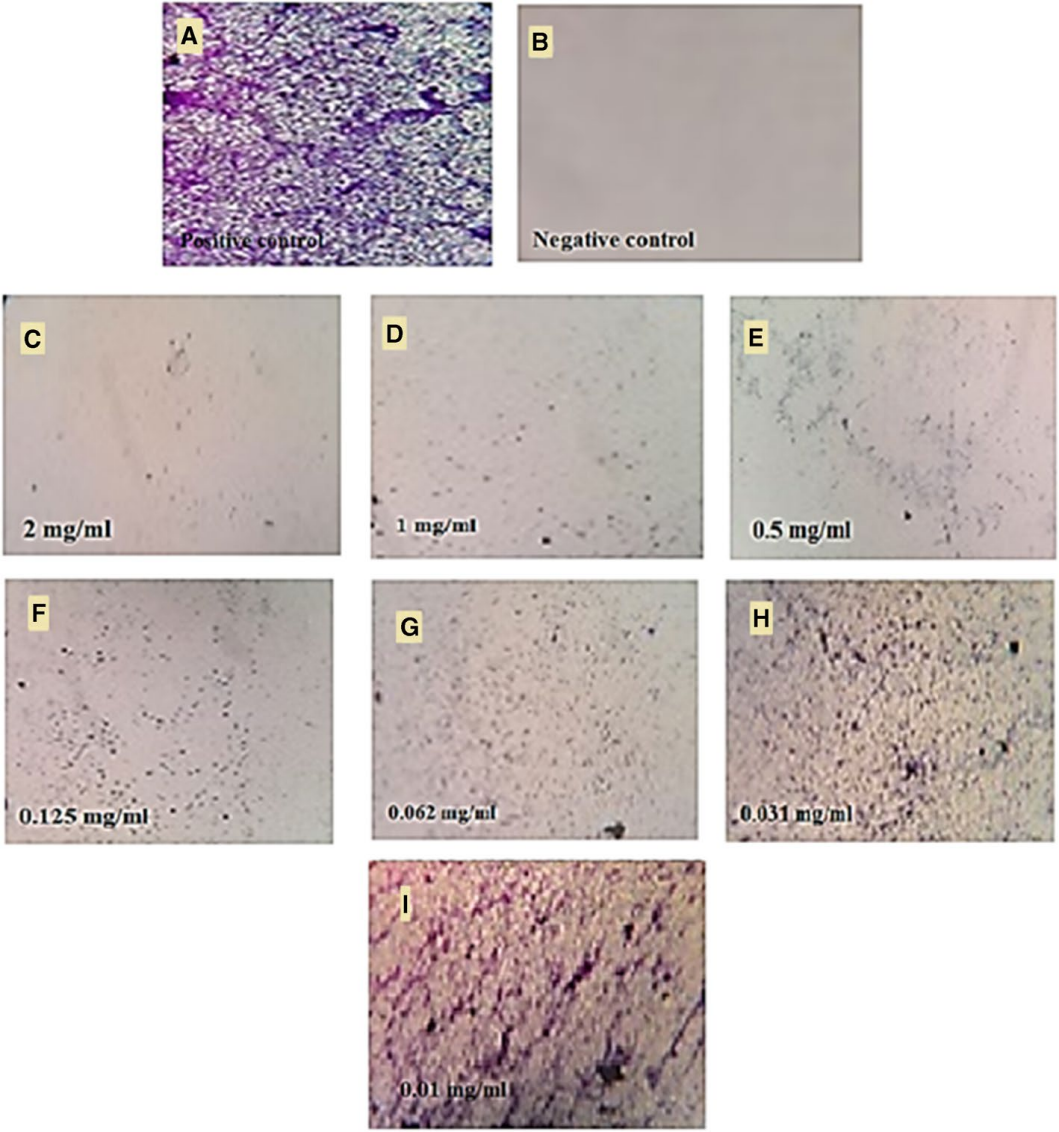
Light inverted microscopic images of *S. aureus* biofilm grown with various concentrations of Au-NPs. 0.0 mg/mL represents the positive control (**A**). Negative control (**B**). 2.0, 1.0, and 0.5 mg/mL (**C**, **D**, **E**) above the MIC value. 0.125 mg/mL (**F**). 0.062 mg/mL (**G**). 0.031 mg/mL (**H**). 0.01 mg/mL (**I**). At concentrations from 0.031 and 0.01 mg/mL (**H** and **I**), bacteria have appeared as scattered cells and cannot aggregate together to perform normal biofilm

In this work, the antibiofilm activity of nanoparticles exhibited varied effects against different microorganisms (Fig. 9). Accordingly, Ag-NPs had a little inhibited effect against *Pseudomonas aeruginosa* and *Staphylococcus aureus* which inhibited up to 44.3% at 31.25 µg/mL, 44.25% at 15.6 µg/mL, and 39.36% at 7.8 µg/mL for *Pseudomonas aeruginosa* (Fig. 9A) and only 57.5% at 125 µg/mL for *Staphylococcus aureus* (Fig. 9B). In contrast, Au-NPs exhibited the highest effect against biofilm formation of *Pseudomonas aeruginosa* and *Staphylococcus aureus* without affecting the bacterial growth when performed at concentrations under MIC value. Au-NPs at 250, 125, 62.5, 31.25, and 15.62 µg/mL had reduced the biofilm formation by 93.6, 86.8, 80.4, 77.5, and 59.4%, respectively against *P. aeruginosa* (Fig. 9C). Furthermore, Au-NPs displayed strong biofilm prevention agent against *S. aureus* at concentrations below the fatal dose without influencing bacterial growth, with proportions of 81.3, 77.4, and 63.0%, respectively (Fig. 9D). The influence of nanoparticles on the surface topology of the biofilm matrix was studied using inverted microscopy analysis. The results revealed the existence of biofilm matrix in the positive-control sample, but Au-NP-treated wells demonstrated decreased surface colonization and biofilm matrix in *S. aureus*. The light microscopic pictures revealed that the Au-NPs had accomplished complete biofilm dispersal by dissolving the micro-colonies inside the specimen allowed to treat in 2000 to 0.015 µg/mL (Fig. 10). Respectively, quantitative and qualitative evaluations demonstrate that Au-NPs decreased *Pseudomonas aeruginosa* and *S. aureus* biofilm formation throughout the first stage. Another study found that AuNPs produced also with fungal strain *Laccaria fraterna* reduced biofilm growth in almost the same way. Our results were likewise phenotypically like those of Rajkumari et al. [88]. Utilizing baicalin-conjugated nanoparticles, they contributed to the reduction of biofilm-forming capability. The results of our crystal violet technique for biofilm inhibition corresponded to those of Khan et al. [89]. Estevez et al. showed that Ag-NPs can diffuse into the matrix and damaged cells in the biofilm’s internal layer [90].

### Cytotoxicity of Ag-NPs and Au-NPs

**Fig. 11:**
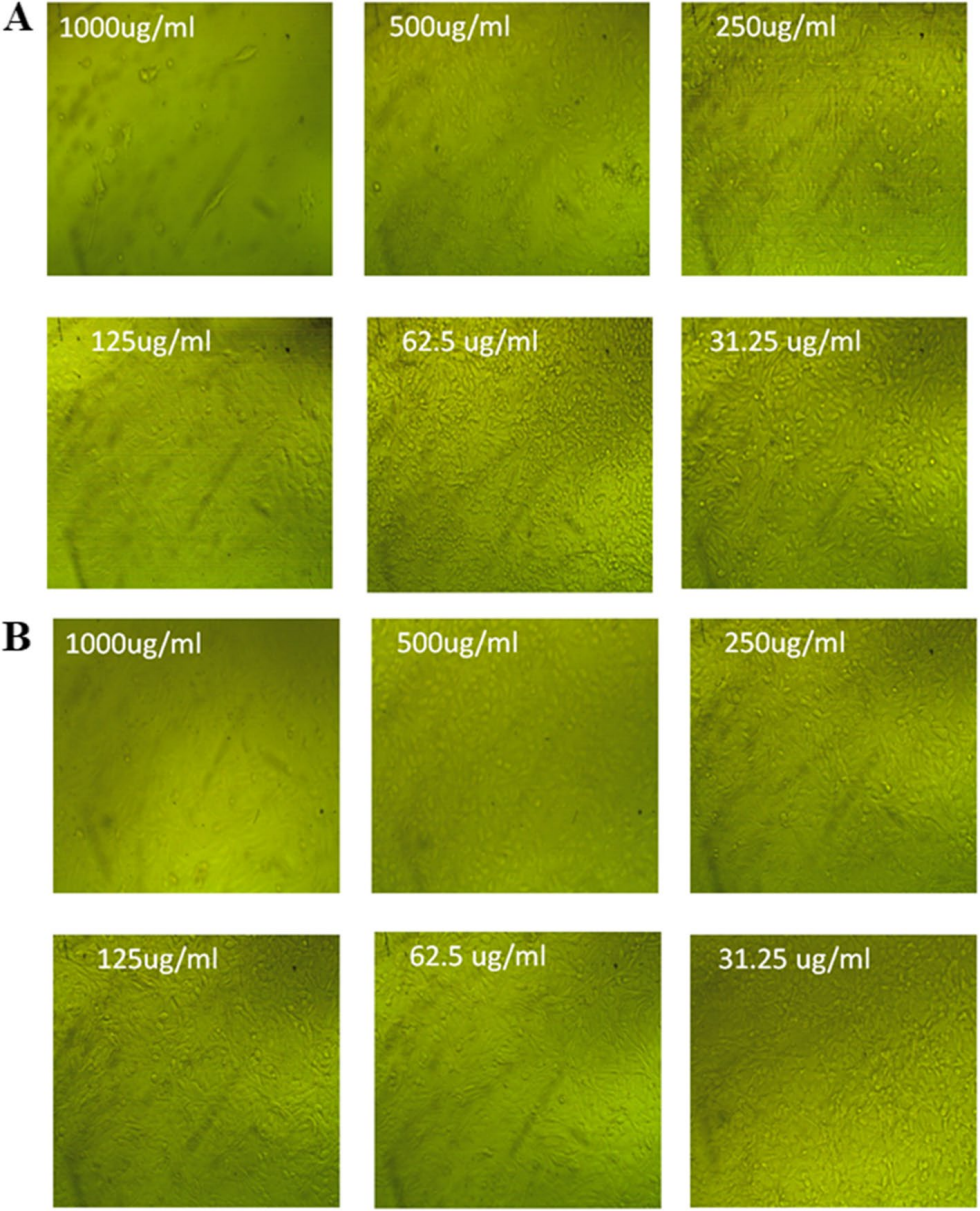
Effect of Ag-NPs (**A**) and Au-NPs (**B**) on normal Vero cells at different concentrations imaged by a light inverted microscope

**Fig. 12:**
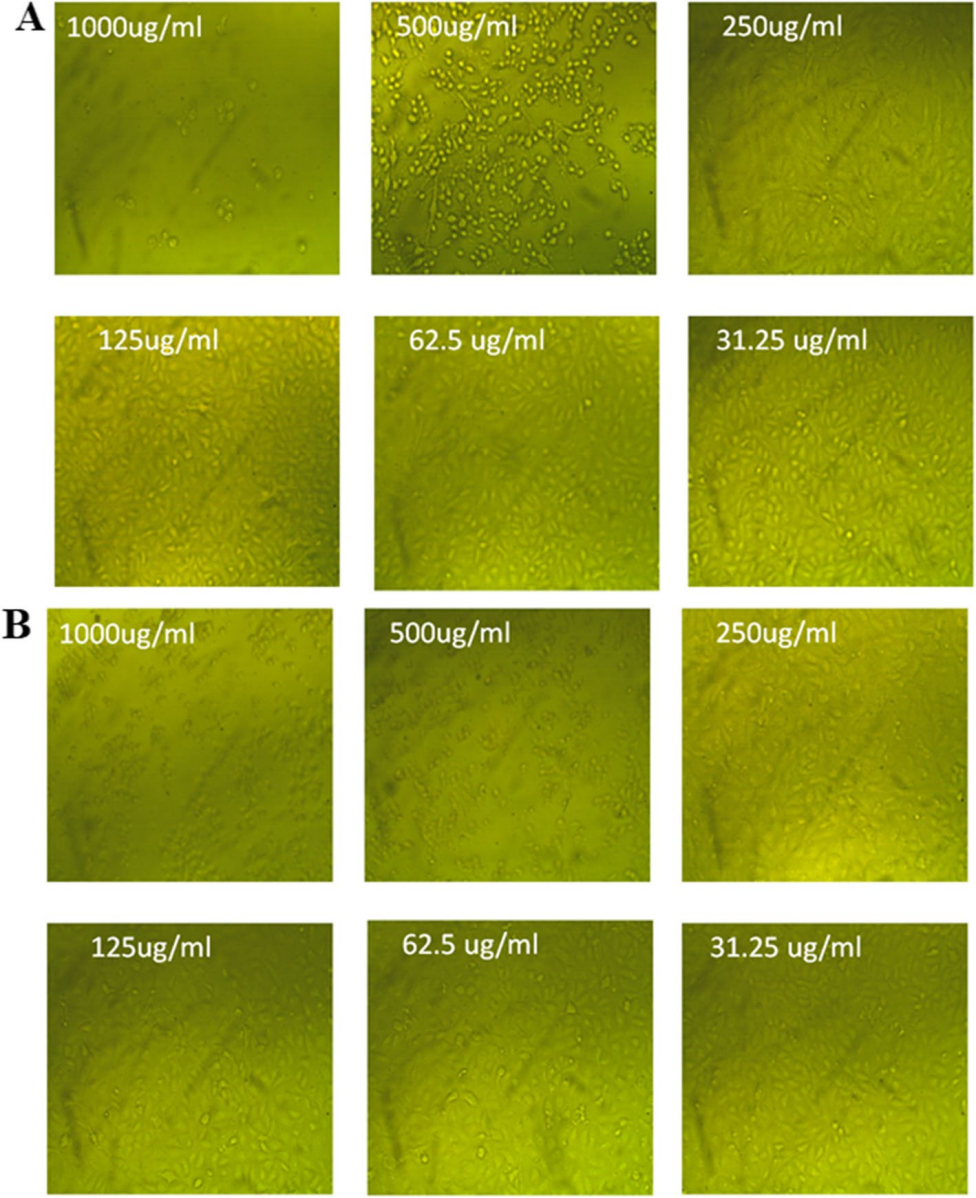
Effect of Ag-NPs (**A**) and Au-NPs (**B**) on cancer cells (Mcf7) at different concentrations imaged by a light inverted microscope

In the shape of the cells, the first and most noticeable finding arising from exposure to nanoparticles or other toxic compounds is a change in cell shape and otherwise morphology of the cell in culture. As a result, the light inverted microscope may be developed to monitor damage to cellular shape and morphology as a result of Ag-NPs and Au-NPs exposed dosage. The normal cell line expanded continuously across the plates and displayed epithelial shape. Cells after treatment to various concentration of Ag-NPs or Au-NPs eventually lost unique phenotypic characteristics. Additionally, at high concentrations of Ag-NPs and Au-NPs, the cells exhibit full or partial breakdown of monolayer, cell granulation, rounding, or shrinkage as compared to control sample. The light inverted microscope picture demonstrated unequivocally that the damage induced in cell morphology is dosage dependent for Ag-NPs and Au-NPs (Figs. 11 and 12).

### MTT Assay

**Fig. 13:**
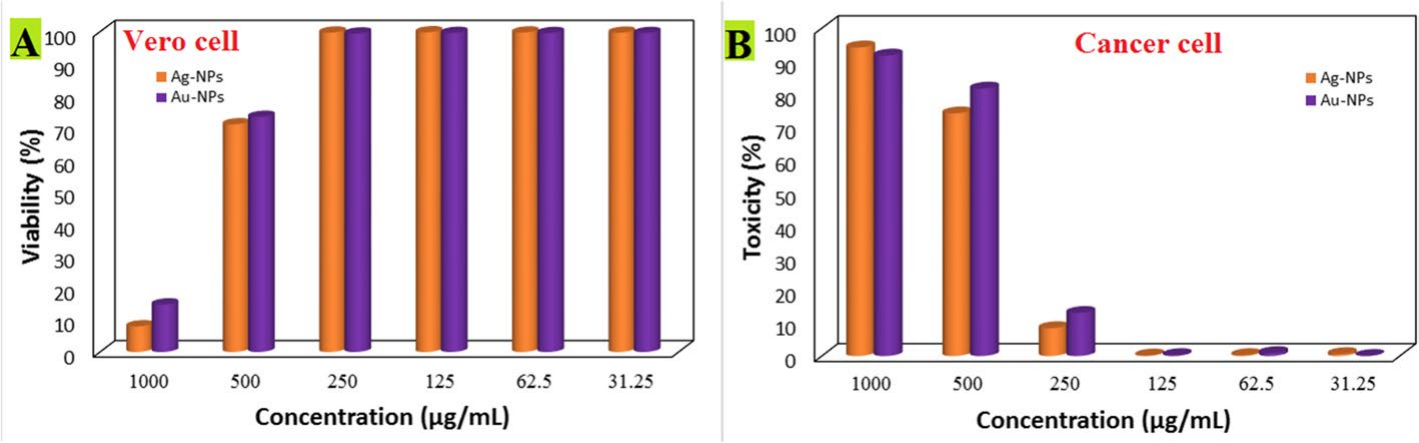
Cytotoxicity (**A**) and antitumor activity (**B**) of Ag-NPs and Au-NPs

Viability assays are a basic stage in toxicology that explain the cellular response to a toxicant. Also, they give information on the cell death, survival, and metabolic activities [91]. MTT assay is a sensitive colorimetric assay for the determination of the number of viabilities in cell proliferation and cytotoxicity assays. Cell mortality for normal and cancer cells was likewise dose dependent when exposed to varied concentrations of Ag-NPs and Au-NPs, as shown in Fig. 13. Particularly, the IC_50_ for normal cell represented by kidney Vero cell was 693.68 μg/mL and 661.24 μg/mL, for Ag-NPs and Au-NPs, respectively (Fig. 13A), while IC_50_ for cancer cell (Mcf7) was 370.56 μg/mL for Ag-NPs and 394.79 μg/mL for Au-NPs (Fig. 13B). Thus, the treatments by Ag-NPs and Au-NPs are highly recommended to be performed at a concentration lower than 693.68 μg/mL and 661.24 μg/mL, respectively, in order to preserve the safety to humans. Several groups have examined the cytotoxicity of Ag-NPs and Au-NPs using various human cancer cells [92, 93]. A similar study revealed the anticancer effect of Ag-NPs and Au-NPs towards HCT-116 carcinoma cells. The IC_50_ value for Ag-NPs and Au-NPs was found to be 100 and 200 μg/mL, respectively [93]. Hamed and his colleagues demonstrated anticancer efficacy against two different cancer cell lines colon carcinoma cells (HCT-116) and breast carcinoma cells (MCF-7) by using Au-NPs for medicinal applications [94].

### Antioxidant Activity

**Fig. 14:**
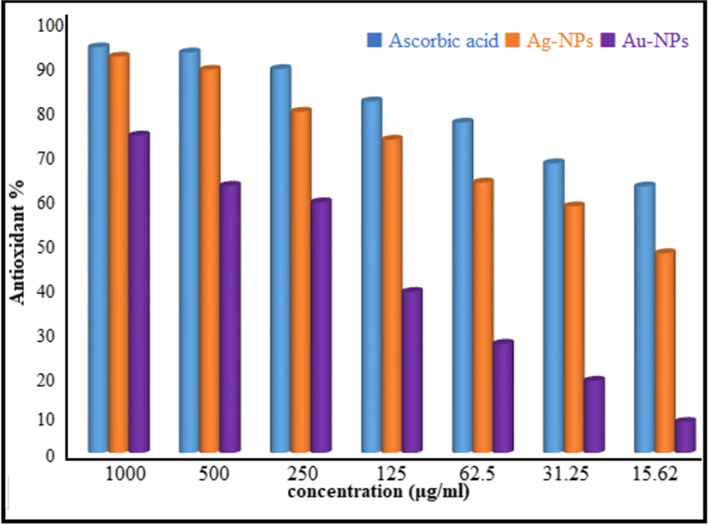
Antioxidant activity of Ag-NPs and Au-NPs by the DPPH method

Reactive oxygen species (ROS), which are by-products of cellular processes, are defeated by antioxidants. Antioxidants can also neutralize free radicals that are responsible for a number of disorders [95]. When antioxidants exhibit antitumor, antibacterial anti-inflammatory, anti-tumor, anti-carcinogenic, anti-mutagenic, and anti-atherosclerotic, they have already been thought of as therapeutic agents. In this work, the antioxidant activity of Ag-NPs and Au-NPs was assessed using DPPH techniques at various concentrations (1000–7.81 μg/mL), as shown in Fig. 14. Results revealed that Ag-NPs had the strongest antioxidant activity compared to Au-NPs. Ag-NPs had an IC_50_ of 19.7 μg/mL as opposed to 194.0 μg/mL for Au-NPs. According to research by Pu et al., spherical Au-NPs have stronger antioxidant potential than irregularly or polygonal ones [96]. In previous research, Ag-NPs showed strong DPPH efficacy with just an IC_50_ value of 30.04 μg/mL [97]. Our results confirm the outstanding antioxidant properties of Ag-NPs and Au-NPs that have been previously described [98, 99].

## Conclusion

In the current study, the mycosynthesis of Ag-NPs and Au-NPs were performed by isolated soil fungus *Trichoderma saturnisporum*. Complete characterization of Ag-NPs and Au-NPs was carried out using UV–vis, FTIR, XRD, TEM, and SEM in which both NPs were spherical in shape and size ranging from 10 to 70 nm for Ag-NPs and 8–30 nm for Au-NPs. It is feasible to assume that the substances (proteins) contained in mycelial-free filtrate play a significant role in the reduction and stability of Ag-NPs and Au-NPs based on the results of the FTIR study. Gram-positive and Gram-negative bacteria, antibiofilm, and other microorganisms were all successfully inhibited by the two metallic nanoparticles when used in biological applications. Additionally, they demonstrated potent antioxidant and anticancer action against a breast cancer cell line without having any negative effects on normal cell lines (Vero). Therefore, these nanocompounds produced by this soil fungus may be considered safe and can become alternatives to commercial antimicrobial agents.

## Data Availability

The data used to support the findings of this study are available from the corresponding author upon request.
